# The Influence of Implant Surface Modification on Marginal Bone Loss and Periodontal Health: A Cross-Over Randomized Clinical Trial

**DOI:** 10.1155/ijod/8889144

**Published:** 2025-09-28

**Authors:** Beatrice Longhi, Francesco Pera, Maria Menini, Francesco Bagnasco, Paolo Pesce, Marino Caroprese, Giuseppe Troiano, Khrystyna Zhurakivska

**Affiliations:** ^1^Department of Oral Rehabilitation and Maxillofacial Prosthesis, Dental School, University of Turin, Turin, Italy; ^2^Department of Surgical Sciences and Integrated Diagnostics, Unit of Implant and Prosthetic Dentistry, University of Genoa, Genova, Italy; ^3^Department of Clinical and Experimental Medicine, University of Foggia, Foggia, Italy; ^4^Department of Medicine and Surgery, LUM University, Casamassima, Italy

**Keywords:** anodized collar, MBL, short implants, surface treatment

## Abstract

**Background:** Edentulism rehabilitation with short implants is a procedure of proven efficacy. To improve the biological aspects of the interface between the implant and hard and soft tissues, different implant and prosthetic surface treatments have been proposed, producing contrasting results. The aim of present study is to compare implants and transmucosal components with an anodized collar with those with a traditionally machined collar in terms of Marginal bone loss and periodontal indexes.

**Materials and Methods:** 30 patients were treated with two adjacent 6 mm length and 4.3 mm diameter implants (Shard short, Mech&Human, Grisignano di Zocco, Italy), one with an anodized collar (Test group) and one with a traditional machined collar (Control group), randomly positioned. Definitive transmucosal straight multiunit abutments (MUAs) (Mech&Human, Grisignano di Zocco, Italy) of height 1 mm, with differentially treated surfaces, were immediately screwed. After 3 months, prosthetic rehabilitation with splinted zirconia crowns screwed to the MUAs was made. Marginal bone levels (MBLs) were evaluated at the time of implant placement (T0), after 3 months (T3), after 6 and 12 months (T6 and T12) through periapical radiographies. Periodontal indexes (probing depth [PD], bleeding on probing [BoP], and plaque index [PlI]) were evaluated at the same timepoints, with the maximum follow-up of 12 months.

**Results:** Average marginal bone loss at T3 was 0.40 ± 0.31 mm in the Test group and 0.42 ± 0.40 mm in the Control group (*p*=0.76), reaching 0.63 ± 0.41 and 0.78 ± 0.43 mm at T12 in the Test and the Control groups, respectively (*p*=0.94). Physiological PDs, with average values ranging between 1.48 and 2.1 mm, were revealed around the implants in both the groups, and The PlI ranged between 0 and 1 in most cases, and BoP appeared in some cases with isolated bleeding spots after probe passing (mean values ranging between 0.20 ± 0.41 and 0.50 ± 0.52), with no significant differences between groups.

**Conclusions:** Surface treatment with anodization of implant collar and transmucosal components seem to not influence marginal bone stability at 1-year follow-up, nor the condition of periodontal tissues. Long-term follow-ups are needed to confirm the results.

**Trial Registration**: ClinicalTrials.gov identifier: NCT05766878

**Summary**

• What is known: Dental implants with treated surfaces have been proposed to improve their biological interaction with surrounding tissues. Nevertheless, their clinical performance is still controversial.
• What this study adds: This study suggests that implants with anodized collar and anodized transmucosal components, when compared to those with a traditionally machined surface, produce similar results in terms of marginal bone loss and periodontal indexes.

## 1. Introduction

Dental implant placement has become a widely accepted procedure for rehabilitation of partially and fully edentulous patients. The long-term success of implant therapy depends on various factors, including implant characteristics, surgical and prosthetic techniques, and patient-specific conditions [1–4]. Availability of sufficient bone structure is essential for implant placement. The modifications of alveolar socket, following tooth extraction, can lead to extensive bone reabsorption, making the implant procedure more complicated [5]. Jaw atrophy can represent a challenge for implant procedures, especially in the posterior maxilla and mandible, where anatomical constraints, such as the maxillary sinus and inferior alveolar nerve, can limit standard implant placement. To overcome these drawbacks, additional surgical techniques may be necessary, such as bone augmentation or sinus floor elevation [6, 7]. Such procedures, although well documented and standardized, carry risks inherent to the surgical intervention, and various complications may occur [8, 9].

Short implants have been introduced with the aim of avoiding advanced surgical procedures in cases where vertical bone availability is limited. With the advancement of knowledge about the micro and macro structure of the fixture, short implants became a reliable solution, as an alternative to bone grafting procedures, offering reduced surgical complexity, lower morbidity, shorter treatment time, and decreased costs [10, 11].

The characteristics of implant surface play a crucial role in osseointegration and long-term stability. Various surface treatments have been developed to enhance the biological response of peri-implant tissues [12]. Among these, anodization has been proposed for altering the oxide layer thickness and surface porosity, which may improve osteoconductive properties and influence osseointegration process [13–15]. The effects of surface anodization on peri-implant soft tissues have also been investigated. Some histological analyses revealed increased blood vessel proliferation and lower inflammatory infiltrate in peri-implant tissues adjacent to anodized surfaces, compared to machined compounds [16, 17].

Alongside these potential benefits; however, an increased roughness has been supposed to promote bacterial adhesion and make the oral hygiene practice less effective in plaque removal, leading to a growing risk of progressive tissue inflammation and development of peri-implant diseases [18–20].

The literature is heterogeneous on this aspect, reporting in some cases a worse survival of anodized implants, when compared to sandblasted large-grit acid-etched ones, but with other overlapping clinical results [21]. To limit these possible drawbacks, the treatment of the only implant collar has been proposed, showing encouraging results in terms of cellular response [22, 23]. However, the results obtained from in vitro tests do not yet present sufficient clinical evidence. Several histological studies have been conducted using animal models, but the conclusions are not univocal in affirming an advantage of the treated surfaces of the transmucosal components, compared to the untreated ones [24]. Few clinical data are available in the literature, suggesting negligible clinical effects of anodized abutment collars on peri-implant soft tissues, compared to the untreated surfaces [25].

The aim of present randomized clinical trial was to prospectively evaluate the performance of short implants with different surface characteristics. Specifically, comparing implants and transmucosal components with an anodized collar (Test group) to those with a traditionally machined collar (Control group) in terms of Marginal bone loss and periodontal indexes. The null hypothesis is that no differences in investigated parameters were present between the two types of collars and transmucosal surface treatments.

## 2. Materials and Methods

### 2.1. Study Design

This multicentric cross-over randomized controlled trial has been conducted following the principles established by the Declaration of Helsinki on experimentation involving human subjects. All patients have been thoroughly informed about the intervention and signed a written consent form. The research has been authorized by the INTERCOMPANY ETHICS COMMITTEE of A.O. City of Health and Science of Turin: A.O. Mauriziano Order of Turin: A.S.L. City of Turin, (N°0069632 of 06/06/2023). Recording on ClinicalTrials.gov has been performed, and N°NCT05766878 has been assigned. CONSORT (Consolidated Standards of Reporting Trials) guidelines have been followed in reporting the study [26].

### 2.2. Patient Selection

All patients needing rehabilitation of posterior maxilla or mandible with two adjacent implants were recruited for potential inclusion in this study. Inclusion criteria were: age ≥ 18 years old; good general health; at least 6 months passed after tooth loss/extraction; reduced ridge height that does not allow placement of long implants; and bone tissue thickness of at least 7 mm. Exclusion criteria were: general medical contraindications to implant placement [27]; past or present antiresorptive treatment; uncontrolled diabetes; pregnancy; and sites subjected to bone regeneration or maxillary sinus lift.

The study took place in three experimental centers: University of Genoa (Genoa, Italy), University of Foggia (Foggia, Italy), and the University of Turin (Turin, Italy), in the period from March 2023 to January 2024.

### 2.3. Surgical and Restorative Protocol

**Figure 1:**
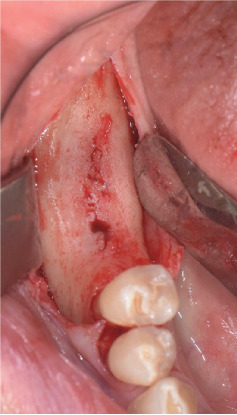
Full-thickness muco-periosteal flap preparation.

All patients received an oral administration by antibiotic prophylaxis of 2 g of amoxicillin, 1 h before surgical treatment. Hence, a local anesthesia was performed, and a full-thickness muco-periosteal flap was raised (Figure 1).

**Figure 2:**
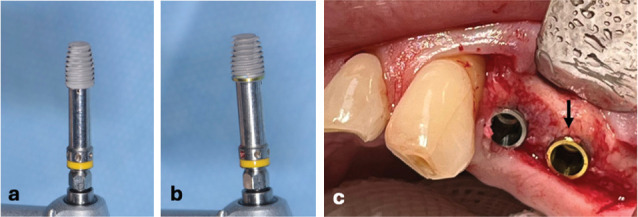
6 mm length and 4.3 mm diameter implants (Shard short, Mech&Human, Grisignano di Zocco, Italy) (a) without and (b) with anodized collar (a, b) before and (c) after placement (the Test implant indicated by the arrow).

Implant site preparations were performed following manufacturer's indications, and two adjacent 6 mm length and 4.3 mm diameter implants, with an average surface roughness of 0.65 μm (Shard short, Mech&Human, Grisignano di Zocco, Italy), were randomly placed in the same quadrant. Both the implants presented surfaces treated with double acid etching. The only difference between the two implants was the surface treatment of the coronal collar (0.3 mm), presenting an anodized collar (Test group) and a traditional machined collar (Control group).  Figure 2 shows the two different implants before (Figure 2a,b) and after placement (Figure 2c).

**Figure 3:**
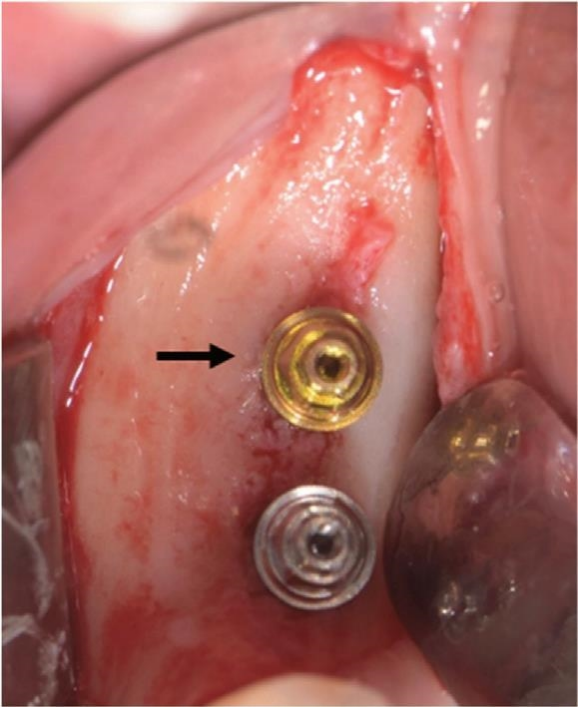
Straight multiunit abutments (MUAs) (Mech&Human, Grisignano di Zocco, Italy) with anodized surface (indicated by the arrow) and standard machined surface, screwed on the Test and the Control implant, respectively.

Insertion torque (Ncm) was recorded for each implant. Definitive transmucosal straight multiunit abutments (MUAs) (Mech&Human, Grisignano di Zocco, Italy) of height 1 mm were immediately screwed: a MUA with anodized surface on the Test implant and a standard machined MUA on Control implant (Figure 3). The MUAs were tightened to 25 Ncm, according to the one-abutment one-time approach. The anodization process of transmucosal components (implant collars and MUAs) was performed using sodium bicarbonate (NaHCO_3_) as the electrolyte.

A single stitch suture was placed, and the flap was fitted around the trans-epithelial abutments. If the patient did not suffer from systemic diseases that alter the immune system, no antibiotic therapy was prescribed. The use of 0.2% chlorhexidine mouthwash twice daily was recommended. The use of painkillers was recommended as needed. 10 days after, the sutures were removed and postsurgical sites were left to an unsubmerged healing for 3 months, without any temporary prosthetic rehabilitation.

**Figure 4:**
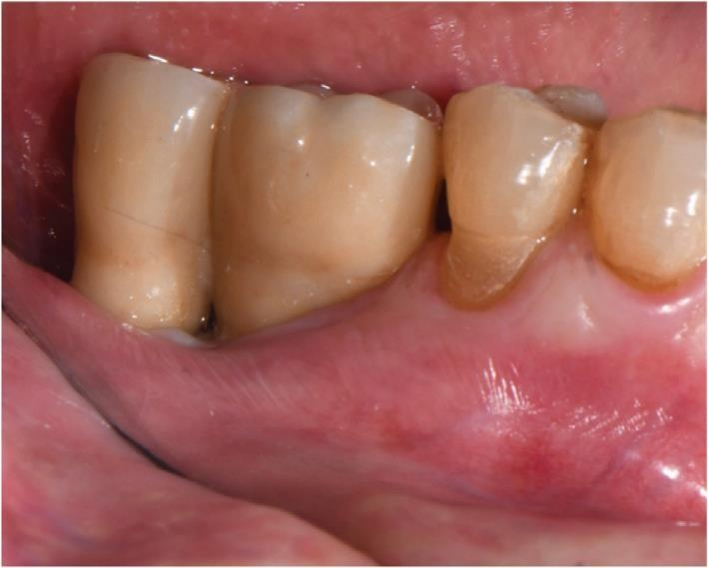
Splinted zirconia crowns, screwed to the MUAs.

After 3 months, abutment-level intraoral scans were performed, and prosthetic rehabilitation was made with placement of splinted zirconia crowns, screwed to the MUAs (Figure 4).

### 2.4. Outcome Measures

**Figure 5:**
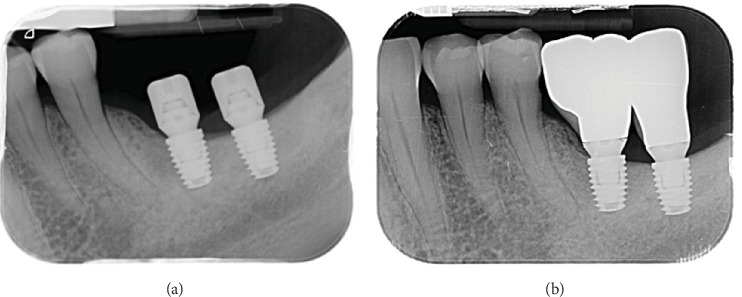
Periapical radiographs performed with paralleling technique and a customized bite jig taken at (a) 3 and (b) 6 months follow-up.

The primary outcome regarded the measurement of marginal bone level (MBL) changes observed after implant placement and prosthetic rehabilitation. For this purpose, radiographic evaluations were made using periapical radiographs performed with the paralleling technique and a customized bite jig. MBL evaluations were made at the following time points: at implant placement (T0), 3 months after placement and before prosthetic rehabilitation (T3), six (T6), and 12 (T12) months after implant placement (Figure 5a,b).

Peri-implant MBLs were measured mesially and distally to the implants, at each time point as the linear distance between the implant platform and the edge of the bone crest (mm). The measurements were performed using a measuring software (Image J 1.52a, National Institutes of Health, Bethesda, Maryland) with calibration of each image referred to the known implant length. Each measurement was independently performed by two examiners (Beatrice Longhi and Khrystyna Zhurakivska) and repeated three times by each other, as suggested by Gomez-Roman and Launer [28]. The examiners were blinded to the Test and Control implants on the radiographies. Intraexaminer and interexaminer agreement, expressed by Cohen's *k* coefficient, was 0.813, respectively, for linear measurements within ± 0.1 mm. Marginal bone loss was then calculated as the difference in MBLs detected at different timepoints.

Secondary endpoints were represented by periodontal evaluations. In particular, the following periodontal indexes were assessed:

– Probing depth (PD): evaluated as linear distance, expressed in millimeters, from the gingival margin to the bottom of the gingival sulcus or periodontal pocket, measured using a periodontal probe at six sites per implant;
– Plaque index (PlI): defined by Silness and Loe [29] and expressed on a scale from 0 to 3, where 0 indicates no plaque and 3 reflects abundance of soft matter within the gingival sulcus and/or on the prosthetic compounds and gingival margin. PlIs were revealed at four surfaces of each implant (mesial, distal, buccal, and palatal/lingual) at different time points. The mean value was then considered for statistical analysis.
– Bleeding on Probing (BoP) was evaluated using the revised papillary bleeding index [30] around each implant, expressing the condition with a scale going from 0 (no bleeding) to 4 (spontaneous bleeding or profuse bleeding that occurs without probing or upon very slight manipulation).

### 2.5. Sample Size and Randomization

Sample size was calculated using web-based software (https://app.sampsize.org.uk). Effect size was calculated based on a clinically meaningful difference in MBL after 6 months from placement around 6-mm short implants of 0.25 mm, with a population standard deviation reported in a previous study of 0.21. Considering the cross-over design, the most conservative scenario was assumed by setting the intrasubject correlation (*ρ*) to 0, which results in the largest required sample size [31]. Under these assumptions, a minimum of 22 patients (44 implants) would be required to detect a significant difference with a two-tailed α of 1% and a power of 90%. To account for potential dropouts (estimated at 20%), the final planned enrollment was set at 30 patients (60 implants).

A computer-generated table was prepared using a balanced, randomly permuted block approach (www.random.org), allocating the two implants of each patient to the specific group (Test and Control), with a block size of four. Randomization lists were generated separately for each of the three experimental centers. Patients were randomly assigned to receive either the Test or Control implant first, assuring an even distribution across the study population. Randomization codes had been hidden until the flap reflection. The surgeon was blinded during the preparation of the first site until the implant fixture was inserted. Radiological measurements of changes in MBL were performed blindly by the investigator. The yellow color of the anodized components prevented complete double blinding during the clinical phases (implant insertion and periodontal index measurement).

### 2.6. Statistical Analysis

Data analysis was performed by an independent investigator using STATA 16.0 software (StataCorp, College Station, Texas). Descriptive statistics were calculated as frequencies for categorical variables and means with standard deviations for continuous variables. Evaluation of normal distribution in the assessed outcomes at different time points was performed by means of the Shapiro–Wilk test. Differences between continuous variables between the two groups (Control and Test) were assessed with a *t*-test for parametric data and with a two-sample Wilcoxon rank-sum test for nonparametric data, whereas the *χ*^2^ test was used to evaluate categorical variables. A multivariate linear regression models were built in order to test the influence of implant positioning in different jaws (maxilla/mandible) on the marginal bone loss at different time points.

## 3. Results

Recruitment and interventions were carried out in three experimental centers, including dental clinics at: theUniversity of Genoa (Genoa, Italy), the University of Foggia (Foggia, Italy), and the University of Turin (Turin, Italy), in the period from March 2023 to January 2024. After reaching 30 patients (10 in each center), as per sample size calculation, the enrollment was suspended. All the patients completed the treatment and follow-up visits. The age of the patients ranged between 24 and 82 years, with a mean of 6003 ± 1263 years. The sample was equally distributed between males (14 patients) and females (16 patients). Each patient was treated with two adjacent implants (60 implants), placed in the posterior maxilla (18 implants) or mandible (42 implants). A summary table with general characteristics of the sample is reported in Table 1.

### 3.1. Primary Outcomes

At T3, all the implants resulted osseointegration, and no biological complications were reported and recorded. After prosthetic rehabilitation, at each following time point (T6 and T12), 56 implants were in good function. Four implants, two in the maxilla and two in the mandible, reported a prosthetic complication due to the unscrewing of the connecting screw. This complication equally affected two implants with an anodized collar and two with an untreated surface. No other biological or mechanical complications were observed.

MBLs of the investigated groups were compared using a two-sample Wilcoxon rank-sum test, with no significant differences revealed at any time point (Table 2).

On average a marginal bone loss of 0.40 ± 0.31 mm was observed in the Test group and 0.42 ± 0.40 mm in the Control group between T0 and T3 (*p*=0.76). The extension of bone loss increased to 0.51 ± 0.51 mm at T6 and 0.63 ± 0.41 mm at T12 in the Test group and 0.53 ± 0.46 mm at T6 and 0.78 ± 0.43 mm at T12 in the Control group. All the differences between groups were not significant, with *p*=0.90 and 0.94 at T6 and T12, respectively. However, intragroup analysis revealed statistically significant differences between MBLs measured at T3, T6, and T12, compared to baseline. This occurred for both the Test and Control groups (Table 3). No significant differences in primary outcome emerged between maxilla and mandible at T3 (*p*=0.829), T6 (*p*=0.747), and T12 (*p*=0.434).

### 3.2. Secondary Outcomes

Periodontal indexes revealed good periodontal conditions around the implants at each time point. The PlI, in most cases, ranged between 0 and 1, with an average value of 0.45 ± 0.44 in the Test group and 0.51 ± 0.42 in the Control group (*p*=0.54) at T3, 0.64 ± 0.47 and 0.58 ± 0.51 (*p*=0.65) at T6, 0.57 ± 0.47 and 0.52 ± 0.50 (*p*=0.78) at T12 in the Test and Control group, respectively. Physiological PDs were revealed around the implants. The average measures in the Test and the Control groups were: 1.48 ± 0.80 and 1.56 ± 0.56 mm at T3 (*p*=0.49), 1.88 ± 0.83 and 2.1 ± 0.78 mm at T6 (*p*=0.37), 2.14 ± 0.80 and 2.01 ± 0.85 mm at T12, respectively. Only one patient reported an increased PD, reaching 5 mm in different surfaces of implants. No patients reported spontaneous bleeding or suppuration. BoP was revealed around 12 implants at T3, 13 implants at T6, and 10 implants at T12, manifesting with isolated bleeding spots after probe passing around the gingival margin. No significant differences in periodontal indexes emerged between the groups (Table 4). Furthermore, in the comparison between the mandible and maxilla, the secondary outcome values were similar, with no statistically significant differences.

## 4. Discussion

The present study aimed to compare the performance of short dental implants with different collar surface treatments, specifically anodized versus traditionally machined collars, in terms of marginal bone loss and periodontal health. The cross-over design of the study allowed to minimize the influence of patient-related confounding factors, with the same patient used as Test and Control. The results demonstrated that both implant types performed similarly, with no significant differences in terms of marginal bone loss or periodontal parameters across the follow-up period.

In particular, marginal bone loss around Test implants was 0.40 mm 3 months after placement, 0.51 mm 6 months after placement, and 0.63 mm 1 year after implant placement. The Control implants presented similar values, with no significant differences at any timepoint (*p*=0.76; *p*=0.90; and *p*=0.94). The values detected are in line with those reported in other studies investigating bone remodeling around implants and can be considered largely within the range of physiological remodeling that follows implant insertion and its prosthetic loading [32]. Such a range has been investigated and redefined by several authors, claiming that a marginal bone loss of 1.5–2 mm, observed in the first year of functional load, can be considered a good outcome [33–35]. Nevertheless, more recent studies suggest that early bone loss should not exceed 0.5–1 mm in order to limit the risk of developing long-term peri-implantitis [36, 37]. In light of this evidence, the results obtained in this study are encouraging, even if a long-term observation is needed to draw more consistent conclusions.

With reference to the use of short implants, the marginal bone stability becomes even more critical. However, these do not seem to show any differences in terms of marginal bone loss, as well as PD, compared to conventional length implants [38]. These observations seem to have an impact on other parameters, such as implant survival, which appears to be similar between conventional and short implants [39].

Considering atrophic jaws, short implants have been compared with conventional-length implants placed in areas undergoing sinus elevation. In terms of implant survival, marginal bone stability, and complication rates, different studies report comparable results at long-term evaluation [40]. A meta-analysis conducted by Ravidà et al [11]. compared clinical and patient-reported outcomes of short (≤ 6 mm) and long (≥ 10 mm) dental implants. The results revealed fewer biological complications and less marginal bone loss for the short implants, associated with reduced surgical time and treatment costs. However, long implants reported a significantly lower rate of prosthetic complications, especially at the 3-year follow-up [11].

Regarding implants with surface modification, existing evidence states a worse survival rate of anodized implants, compared to the sandblasted large-grit acid-etched implants [21]. The same systematic review reports a similar marginal bone loss in the two groups at short-time follow-up, with values increasing in the anodized group at 5-year follow-up. This leaves the question of MBL on surface-treated implants open and requires long-term evaluation. Nevertheless, some studies included in the cited systematic review [21], report bone loss values considerably higher than those we found, already at 1 year of follow-up, reaching 2.0 mm in the study of Astrand et al. [41] and 1.54 mm in the study of Eliasson. The other included studies report MBL at 1-year evaluation below one millimeter, as in our study [42–44]. For other periodontal parameters, no significant differences between groups are reported [21]. However, it is important to note that all the considered studies included implants treated on their entire surface and not only on the collar. This can significantly influence the behavior of the bone-implant interface. Surface modification of the collar alone has the intent to impact microbial attachment, without influencing osseointegration processes.

Referring to the effects of collar-only modifications on peri-implant tissues, the available studies consider machined and rough collars, microthreads, and laser microtexturing. Some evidences show that machined collar implants have a higher risk of early failure than rough collar implants, with higher bone resorption, while microthreads and Laser microtexturing not seem to reduce bone resorption [45]. Several histological studies have been performed in animal models, evaluating if the surface properties of transmucosal implant components can influence the peri-implant soft tissues. However, no definitive conclusions have been drawn, as shown by Canullo et al. [24] in their systematic review and meta-analysis.

Regarding anodization of transmucosal components, few data are available. In a split-mouth clinical study, the anodization of abutment collars has been investigated for its effects on peri-implant soft tissues. No statistically significant differences have been revealed in terms of PD, plaque accumulation, and bleeding around the anodized group, compared to the unanodized one [25]. The width of keratinized mucosa was not considered in this study, since the implants (Test and Control) were adjacent, so most likely characterized by the same tissue anatomy. Furthermore, according to a recent meta-analysis, the role of keratinized mucosa in influencing the PD and marginal bone loss around implants is not confirmed [2]. The only parameter that seems to be influenced by the quality of soft tissues is the PlI. For this reason, the preservation of soft tissues in atrophic jaws can be relevant for maintaining good hygiene around the implants. Further studies investigating this aspect are needed to draw clearer conclusions.

Another parameter that could influence the condition of peri-implant tissues is the quality of soft tissues around the implants, even if a recent meta-analysis revealed a low impact of keratinized mucosa on PD and marginal bone loss. The width of keratinized mucosa was not considered in this study, since the implants (Test and Control) were adjacent, so most likely characterized by the same tissue anatomy.

In our study, the results obtained from periodontal evaluations did not reveal any significant difference in terms of plaque accumulation, PD, and BoP, showing good peri-implant health in both the groups. This result could be explained by several factors. First, it must be considered that periodontal health is influenced by multiple patient-related factors, which may play a predominant role compared to the chemical-physical characteristics of the implant surface. These include oral hygiene, daily habits, salivary composition, etc. In particular, given that the PlI, in addition to showing no significant differences between the two groups, was consistently low across the various time points, it is likely that the patients' good oral hygiene also positively influenced other periodontal parameters, thus attenuating the potential effect of the chemical treatment of the implant surface. Furthermore, the use of NaHCO_3_ for surface anodization could have produced nanotopographical modifications that are insufficient to alter the biological response or affect microbial adhesion in a clinically relevant way.

The main limitations of this study lie in the length of follow-up, limited to 1 year. A longer follow-up could reveal the emergence of some differences between anodized and untreated implants. A longer follow-up would also allow for a better assessment of the performance of short implants and the possible occurrence of biological and/or mechanical complications. Another limitation lies in the study design, which involved the placement of two adjacent implants. Although the minimum distance of 3 mm between the implants was always respected, their proximity and splinting with crowns could influence the assessed parameters. Furthermore, a larger sample size would increase the reliability of the results and would have allowed a further stratification, in order to investigate the influence of other parameters, such as smoking or implant site, on marginal bone loss and other periodontal parameters.

## 5. Conclusions

Surface treatment with anodization of implant collar and transmucosal components seem not to influence the marginal bone stability, nor the condition of periodontal tissues at 1-year follow-up. Future studies incorporating longer follow-up periods and larger sample sizes are necessary to confirm these preliminary findings and fully elucidate the long-term impact of collar surface treatments on short dental implants.

## Figures and Tables

**Table 1:** General characteristics of the sample.

Patient	*N*
Male/female	14/16
Smokers/nonsmokers	5/25
Mean age (years) ± SD	60.03 ± 12.63
Mandible/maxilla	21/9
Mean torque (Ncm) ± SD	51.59 ± 11.31

**Table 2:** Marginal bone level (MBL) expressed in millimeters (mean ± SD).

Groups	T0 mesial	T0 distal	T0 mean	T3 mesial	T3 distal	T3 mean	T6 mesial	T6 distal	T6 mean	T12 mesial	T12 distal	T12 mean
Test	0.30 ± 0.49	0.21 ± 0.49	0. 26 ± 0.45	0.72 ± 0.47	0.60 ± 0.68	0.66 ± 0.51	0.88 ± 0.42	0.66 ± 0.68	0.77 ± 0.48	1.07 ± 0.34	0.90 ± 0.47	0.99 ± 0.34
Control	0.20 ± 0.60	0.36 ± 0.48	0.28 ± 0.49	0.65 ± 0.68	0.73 ± 0.69	0.70 ± 0.64	0.68 ± 0.73	0.89 ± 0.49	0.81 ± 0.59	0.99 ± 0.51	1.13 ± 0.47	1.06 ± 0.46
*p*-Value	0.54	0.36	0.70	0.95	0.50	0.72	0.38	0.42	0.82	0.68	0.48	0.79

*Note:* Revealed at mesial and distal aspects of each implant and their mean value at different time points.

**Table 3:** Mean marginal bone loss (mm) around each implant at different time points with *p*-values of inter and intragroup analysis.

Groups	T3–T0 mean (*n* = 60)	Intragroup *p*-value	T6–T0 mean (*n* = 60)	Intragroup *p*-value	T12–T0 mean (*n* = 60)	Intragroup *p*-value
Test	0.40 ± 0.31	0.014	0.51 ± 0.51	0.002	0.63 ± 0.41	0.001
Control	0.42 ± 0.40	0.043	0.53 ± 0.46	0.009	0.78 ± 0.43	0.001
Intergroup *p*-value	0.76	—	0.90	—	0.94	—

**Table 4:** Mean values of periodontal indexes.

Groups	PlI T3	PlI T6	PlI T12	PD T3	PD T6	PD T12	BoP T3	BoP T6	BoP T12
Test (*n* = 30)	0.45 ± 0.44	0.64 ± 0.47	0.57 ± 0.47	1.48 ± 0.80	1.88 ± 083	2.14 ± 0.80	0.38 ± 0.63	0.2 ± 0.41	0.25 ± 0.46
Control (*n* = 30)	0.51 ± 0.42	0.58 ± 0.51	0.52 ± 0.50	1.56 ± 0.56	2.1 ± 0.78	2.01 ± 0.85	0.24 ± 0.43	0.38 ± 0.49	0.5 ± 0.52
*p*-Value	0.54	0.65	0.78	0.49	0.37	0.69	0.50	0.21	0.26

*Note:* PD, probing depth (mm).

Abbreviations: BoP, bleeding on probing; PlI, plaque index.

## Data Availability

The datasets generated and analyzed during the current study are available from the corresponding author upon request.

## References

[1] Da Silva D. M., Castro F., Martins B., Fraile J. F., Fernandes J. C. H., Fernandes G. V. O. (2025). The Influence of the Gingival Phenotype on Implant Survival Rate and Clinical Parameters: A Systematic Review. *Evidence-Based Dentistry*.

[2] Ravidà A., Arena C., Tattan M. (2022). The Role of Keratinized Mucosa Width as a Risk Factor for Peri-Implant Disease: A Systematic Review, Meta-Analysis, and Trial Sequential Analysis. *Clinical Implant Dentistry and Related Research*.

[3] Remísio M., Borges T., Castro F., Gehrke S. A., Fernandes J. C. H., Fernandes G. V. O. (2023). Histologic Osseointegration Level Comparing Titanium and Zirconia Dental Implants: Meta-Analysis of Preclinical Studies. *The International Journal of Oral & Maxillofacial Implants*.

[4] Stacchi C., Bassi F., Troiano G. (2020). Piezoelectric Bone Surgery for Implant Site Preparation Compared With Conventional Drilling Techniques: A Systematic Review, Meta-Analysis and Trial Sequential Analysis. *International Journal of Oral Implantology (Berlin, Germany)*.

[5] Schropp L., Wenzel A., Kostopoulos L., Karring T. (2003). Bone Healing and Soft Tissue Contour Changes Following Single-Tooth Extraction: A Clinical and Radiographic 12-Month Prospective Study. *The International Journal of Periodontics & Restorative Dentistry*.

[6] Bouwman W. F., Eijsackers F. A., Bravenboer N., Ten Bruggenkate C. M., Remmelzwaal S., Schulten E. (2025). Long-Term Bone Height Changes After Sinus Floor Elevation With Maxillary or Mandibular Bone Grafts: A Radiological Study. *Clinical Implant Dentistry and Related Research*.

[7] Chandrasekaran D., Chinnaswami R., Malathi N., Jayakumar N. D. (2024). Treatment Outcome of Using Guided Bone Regeneration for Bone Augmentation for the Placement of Dental Implants – A Systematic Review. *Journal of Pharmacy and Bioallied Sciences*.

[8] Katranji A., Fotek P., Wang H.-L. (2008). Sinus Augmentation Complications: Etiology and Treatment. *Implant Dentistry*.

[9] Hernández-Alfaro F., Torradeflot M. M., Marti C. (2008). Prevalence and Management of Schneiderian Membrane Perforations During Sinus-Lift Procedures. *Clinical Oral Implants Research*.

[10] Anitua E., Piñas L., Begoña L., Orive G. (2014). Long-Term Retrospective Evaluation of Short Implants in the Posterior Areas: Clinical Results After 10–12 Years. *Journal of Clinical Periodontology*.

[11] Ravidà A., Wang I.-C., Sammartino G. (2019). Prosthetic Rehabilitation of the Posterior Atrophic Maxilla, Short (≤6 mm) or Long (≥10 mm) Dental Implants? A Systematic Review, Meta-Analysis, and Trial Sequential Analysis: Naples Consensus Report Working Group A. *Implant Dentistry*.

[12] Bakitian F. A. (2024). A Comprehensive Review of the Contemporary Methods for Enhancing Osseointegration and the Antimicrobial Properties of Titanium Dental Implants. *Cureus*.

[13] Wang Q., Zhou P., Liu S. (2020). Multi-Scale Surface Treatments of Titanium Implants for Rapid Osseointegration: A Review. *Nanomaterials*.

[14] Traini T., Murmura G., Sinjari B. (2018). The Surface Anodization of Titanium Dental Implants Improves Blood Clot Formation Followed by Osseointegration. *Coatings*.

[15] Sarvaiya B. B., Kumar S., Pathan M. S. H., Patel S., Gupta V., Haque M. (2025). The Impact of Implant Surface Modifications on the Osseointegration Process: An Overview. *Cureus*.

[16] De Souza V. Z., Manfro R., Teixeira L. N., Elias C. N., Joly J. C., Martinez E. F. (2025). Histological and Immunohistochemical Analysis of Peri-Implant Tissue Regeneration at the Interface of Prosthetic Abutments Treated With Anodizing: A Prospective, Randomized Controlled Clinical Trial on the Early Postoperative Period. *The International Journal of Oral & Maxillofacial Implants*.

[17] Dworan J., Aellos F., Grauer J. A. (2025). Dynamics of Mucosal Integration of Machined Versus Anodized Titanium Implants. *Journal of Dental Research*.

[18] Schmidlin P. R., Müller P., Attin T., Wieland M., Hofer D., Guggenheim B. (2013). Polyspecies Biofilm Formation on Implant Surfaces With Different Surface Characteristics. *Journal of Applied Oral Science*.

[19] Teughels W., Van Assche N., Sliepen I., Quirynen M. (2006). Effect of Material Characteristics and/or Surface Topography on Biofilm Development. *Clinical Oral Implants Research*.

[20] Bermejo P., Sánchez M. C., Llama-Palacios A., Figuero E., Herrera D., Sanz Alonso M. (2019). Biofilm Formation on Dental Implants With Different Surface Micro-Topography: An In Vitro Study. *Clinical Oral Implants Research*.

[21] Wen G., Zhang Y., Xie S., Dong W. (2024). The Influence of Two Distinct Surface Modification Techniques on the Clinical Efficacy of Titanium Implants: A Systematic Review and Meta-Analysis. *Journal of Stomatology, Oral and Maxillofacial Surgery*.

[22] Traver-Méndez V., Camps-Font O., Ventura F. (2023). In Vitro Characterization of an Anodized Surface of a Dental Implant Collar and Dental Abutment on Peri-Implant Cellular Response. *Materials (Basel)*.

[23] Hadzik J., Kubasiewicz-Ross P., Gębarowski T. (2023). An Experimental Anodized Titanium Surface for Transgingival Dental Implant Elements—Preliminary Report. *Journal of Functional Biomaterials*.

[24] Canullo L., Annunziata M., Pesce P., Tommasato G., Nastri L., Guida L. (2021). Influence of Abutment Material and Modifications on Peri-Implant Soft-Tissue Attachment: A Systematic Review and Meta-Analysis of Histological Animal Studies. *The Journal of Prosthetic Dentistry*.

[25] Farrag K. M., Khamis M. M. (2023). Effect of Anodized Titanium Abutment Collars on Peri-Implant Soft Tissue: A Split-Mouth Clinical Study. *The Journal of Prosthetic Dentistry*.

[26] Moher D., Hopewell S., Schulz K. F. (2010). CONSORT 2010 Explanation and Elaboration: Updated Guidelines for Reporting Parallel Group Randomised Trials. *BMj*.

[27] Hwang D., Wang H.-L. (2006). Medical Contraindications to Implant Therapy: Part I: Absolute Contraindications. *Implant Dentistry*.

[28] Gomez-Roman G., Launer S. (2016). Peri-Implant Bone Changes in Immediate and Non-Immediate Root-Analog Stepped Implants—A Matched Comparative Prospective Study up to 10 Years. *International Journal of Implant Dentistry*.

[29] Löe H. (1967). The Gingival Index, the Plaque Index and the Retention Index Systems. *The Journal of Periodontology*.

[30] Saxer U. P., Turconi B., Elsässer C. (1977). Patient Motivation With the Papillary Bleeding Index. *Journal of Preventive Dentistry*.

[31] Sui H., Tang Z., Zhang X., Wei D., Meng H., Han J. (2022). A Prospective, Multicentre Study of 6-mm Short Implants in Posterior Alveolar Bone Supporting Splinted Crowns: A 5-Year Follow-Up Study. *Journal of Clinical Periodontology*.

[32] Galindo-Moreno P., León-Cano A., Ortega-Oller I., Monje A., O′Valle F., Catena A. (2015). Marginal Bone Loss as Success Criterion in Implant Dentistry: Beyond 2 mm. *Clinical Oral Implants Research*.

[33] Papaspyridakos P., Chen C.-J., Singh M., Weber H.-P., Gallucci G. O. (2012). Success Criteria in Implant Dentistry: A Systematic Review. *Journal of Dental Research*.

[34] Roos-Jansåker A. M., Lindahl C., Renvert H., Renvert S. (2006). Nine- To Fourteen-Year Follow-Up of Implant Treatment. Part II: Presence of Peri-Implant Lesions. *Journal of Clinical Periodontology*.

[35] Tarnow D. P., Cho S. C., Wallace S. S. (2000). The Effect of Inter-Implant Distance on the Height of Inter-Implant Bone Crest. *Journal of Periodontology*.

[36] Windael S., Collaert B., De Buyser S., De Bruyn H., Vervaeke S. (2021). Early Peri-Implant Bone Loss as a Predictor for Peri-Implantitis: A 10-Year Prospective Cohort Study. *Clinical Implant Dentistry and Related Research*.

[37] Galindo-Moreno P., Catena A., Pérez-Sayáns M., Fernández-Barbero J. E., O’Valle F., Padial-Molina M. (2022). Early Marginal Bone Loss Around Dental Implants to Define Success in Implant Dentistry: A Retrospective Study. *Clinical Implant Dentistry and Related Research*.

[38] Alqhtani N. R. (2024). Long-Term Clinical Outcomes of Short Implants Versus Conventional Implants in the Posterior Mandible. *Journal of Pharmacy and Bioallied Sciences*.

[39] Pradhan Y., Srivastava G., Choudhury G. K., Sahoo P. K., Padhiary S. K. (2024). Short Implant Versus Conventional Implant in the Posterior Atrophic Maxilla: A Systematic Review and Meta-Analysis. *The Journal of Indian Prosthodontic Society*.

[40] Anitua E., Piñas L., Alkhraisat M. H. (2025). Long-Term Comparative Outcomes of Short Implants Versus Maxillary Sinus Elevation in Posterior Maxilla Rehabilitation. *Dentistry Journal*.

[41] Astrand P., Engquist B., Anzén B. (2004). A Three-Year Follow-Up Report of a Comparative Study of ITI Dental Implants and Brånemark System Implants in the Treatment of the Partially Edentulous Maxilla. *Clinical Implant Dentistry and Related Research*.

[42] Eliasson A., Blomqvist F., Wennerberg A., Johansson A. (2009). A Retrospective Analysis of Early and Delayed Loading of Full-Arch Mandibular Prostheses Using Three Different Implant Systems: Clinical Results With Up to 5 Years of Loading. *Clinical Implant Dentistry and Related Research*.

[43] Gamper F. B., Benic G. I., Sanz-Martin I., Asgeirsson A. G., Hämmerle C. H. F., Thoma D. S. (2017). Randomized Controlled Clinical Trial Comparing One-Piece and Two-Piece Dental Implants Supporting Fixed and Removable Dental Prostheses: 4- to 6-Year Observations. *Clinical Oral Implants Research*.

[44] Aglietta M., Siciliano V. I., Rasperini G., Cafiero C., Lang N. P., Salvi G. E. (2011). A 10-Year Retrospective Analysis of Marginal Bone-Level Changes Around Implants in Periodontally Healthy and Periodontally Compromised Tobacco Smokers. *Clinical Oral Implants Research*.

[45] Messias A., Nicolau P., Guerra F. (2019). Titanium Dental Implants With Different Collar Design and Surface Modifications: A Systematic Review on Survival Rates and Marginal Bone Levels. *Clinical Oral Implants Research*.

